# Prediction of Liver Steatosis and Fibrosis Based on Clinical Variables Using a Large National Survey Database

**DOI:** 10.1155/2023/1791500

**Published:** 2023-05-24

**Authors:** Yanal Alnimer, Touleen Alnimer

**Affiliations:** ^1^Hospital Medicine, Virginia Commonwealth University, Richmond, VA, USA; ^2^Department of General Surgery, University of Jordan School of Medicine, Amman, Jordan

## Abstract

**Background:**

Vibration-controlled transient elastography (VCTA) and controlled attenuation parameter (CAP) are used more frequently to diagnose liver fibrosis and steatosis among nonalcoholic fatty liver disease patients. However, limited robust data are available on the clinical variables strongly related to these disorders and who needs to be referred for screening.

**Methods:**

We used the National Health and Nutritional Examination Survey 2017-2018 database to identify the clinical predictors strongly related to liver steatosis and advanced fibrosis. Baseline comparisons among these groups were made based on widely accepted cutoffs. Linear and logistic regressions were performed to identify the associations between the clinical variables and liver steatosis and fibrosis. We used adaptive lasso regression, gradient-boosted model, and decision trees to determine clinical variables strongly related to these outcomes. A Naïve Byes classifier and decision trees were used to calculate the predicted probabilities of liver steatosis and fibrosis.

**Results:**

32% of our population had evidence of liver steatosis using 294 dB/m as a cutoff. An increase in age, serum triglyceride, and body mass index were associated with a statistically significant increase in liver steatosis; in contrast, females had statistically significantly lower values for liver steatosis by 15 points in the multivariable linear regression model. Serum LDL, smoking, and systolic and diastolic blood pressure are poorly associated with liver steatosis in the adaptive lasso regression. On the other hand, sex, tobacco use, metabolic energy expenditure, and serum triglyceride are the least associated with liver fibrosis based on decision tree analysis and a gradient-boosted model. In decision trees, people with a body mass index above 30 and HbA1c above 5.7 have a 72% likelihood of liver steatosis compared to 14% for people with a body mass index below 30. On the other hand, people with a body mass index above 41 have a 38% likelihood of liver fibrosis.

**Conclusion:**

Body mass index, hemoglobin A1c, serum triglyceride level, sex, and age could provide a good prediction for liver steatosis, while body mass index, blood pressure, platelet counts, hemoglobin A1c, serum LDL, or HDL are highly associated with liver fibrosis and should be used as an initial screening tool prior referral for VCTE/CAP.

## 1. Introduction

Nonalcoholic fatty liver disease (NAFLD) has become a significant public health problem in low- and middle-income countries and Western societies [1]. The point prevalence of the disease varies between 10% and 35% worldwide [2]. The prevalence in the United States is higher among Hispanics, males, older people, and those with diabetes [3]. The disease is a spectrum of pathologies ranging from fatty liver infiltration or steatosis, steatohepatitis, liver fibrosis, and hepatocellular carcinoma. Historically, NAFLD was diagnosed clinically in patients with metabolic syndrome components, including diabetes, hypertension, obesity, and dyslipidemia, along with elevated liver biomarkers. Insulin resistance disturbs fatty acid metabolism and increases hepatic fatty acid production, leading to hepatic steatosis, which acts as a precursor for mitochondrial reactive oxygen radical production and lipid oxidation. NASH occurs when this affects enough mitochondria, which could lead to liver fibrosis. Nonetheless, the interplay seems more complex, and NAFLD could occur in certain genetic conditions without insulin resistance (PNPLA gene mutation) [4]. Moreover, the effect of pioglitazone, an insulin sensitizer, in reversing NAFLD is limited, suggesting that other factors play critical roles in NAFLD and that NAFLD could precede the development of the metabolic syndrome [4].

Liver biopsy and MRI were used frequently to assess the degree of hepatic steatosis. However, the former is limited by its invasiveness and sampling variability, and the latter by its cost. Assessing liver steatosis and fibrosis with controlled attenuation parameter (CAP) and vibration-controlled transient elastography (VCTE), respectively, is more suitable, cost-effective, and relatively easy to perform. Also, it measures the degree of liver steatosis and fibrosis in an area that's 100 times larger than the one obtained by liver biopsy (1 cm in width and 5 cm in depth) [5]. Therefore, VCTE and CAP are becoming more frequently used to evaluate the degree of liver fibrosis and steatosis among alcoholic and NAFLD patients [4].

In the recent meta-analysis by Karlas et al., a CAP cutoff of 294 dB/m had the highest Youden index with the best accuracy in predicting liver steatosis among patients with NAFLD (S0 vs. S1–S3) [6]. Similarly, a liver stiffness value of 8.2 kPa has the highest accuracy in predicting advanced liver fibrosis (≤F2 vs. F3-F4) [6]. In our study, we sought to determine the relationship between different clinical parameters (hemoglobin A1c, triglyceride, LDL, HDL, and body mass index) in addition to age, sex, physical activity, smoking, moderate level of alcohol consumption, and average hours of night sleep, and the values of the CAP and liver stiffness at the previously mentioned cutoffs, using the National Health and Nutritional examination survey in 2017-2018 (NHANS). Also, we looked at the probabilities of NAFLD among the United States population.

Our study uses a national database representative of the United States population to identify the predictors strongly associated with these outcomes among NAFLD patients. Potentially identify new predictors or effect modifiers, calculate the mean values for the CAP among subjects with different predictor values, and calculate the predicted probabilities for developing liver steatosis or liver fibrosis among the United States population. In contrast, Zhang et al. used the database to estimate the prevalence of liver steatosis and fibrosis among the United States population [7]. Our analysis focused on determining the strength of the association of different variables using linear, lasso, and gradient-boosted regressions with liver steatosis and fibrosis, which was not done by the Zhang et al. study. This analysis is fundamental to identifying patients who could benefit most from screening for NAFLD using this modality. Also, we used a CAP cutoff of 294 dB/m, which has higher accuracy in predicting liver steatosis among NAFLD patients compared to the 274 dB/m that was used in their analysis [6, 7].

## 2. Method

We used the 2017-2018 data from the National Health and Nutritional Examination (NHANES) database to measure the association between a variety of clinical variables and the degree of liver steatosis as measured by CAP and liver stiffness as measured by kilopascals (kPa) via VCTE. The following datasets were downloaded from the 2017-2018 NHANES website: patient demographics, results of liver elastography, alcohol consumption, smoking behavior, lipid panels, hours of fasting before the CAP and VCTE testing, average daytime sleeping, blood pressure readings, body mass index, and the degree of physical activity. To evaluate the effect of physical activities with the CAP, we converted the duration of weekly physical activities to weekly metabolic energy expenditure using the following procedure: For vigorous physical activities, we multiply the duration of the weekly physical activity by eight, while we multiply the period of weekly moderate physical activities by four, then we aggregated the moderate and the vigorous weekly metabolic expenditure for each subject. Regarding smoking behavior, we modeled the average number of cigarettes smoked in the last month as a continuous variable. All predictors' values approximated the normal distribution without transformation except for the metabolic energy expenditure, which was transformed into a 10-base logarithmic form to approximate the normal distribution.

Datasets were combined using the Full Join command in R-statistical software without excluding any subjects before creating the survey object. Seventy patients with a history of viral hepatitis were excluded from our analysis. Also, we excluded 236 people who did not have ten valid measurements or whose IQR/M > 30%. We used the examination weights (MEC) in our survey regression analysis as recommended by the NHANES website. Fasting weights were added to the examination weight whenever the analysis included a lipid panel. All statistical analyses were done using R version 3.6.2. Continuous baseline values were reported as a median and interquartile range, while categorical variables were reported as proportions with 95 confidence intervals (CI) using the logit function.

Several analyses were done. In the first one, we examined the association between the CAP and the following covariates: patient's age, sex, LDL values, triglyceride values, hemoglobin A1c, body mass index, smoking, weekly metabolic energy expenditure, alcohol consumption, hypertension, hours of fasting before the procedure, and average sleeping hours per day using a linear regression model. Before running the regression analysis, we looked at the association between the outcome (CAP) and each continuous variable using the Loess smoother function to ensure a linear or near-linear association. To have more interpretable estimates in the linear regression model, we divided the low-density lipoprotein and triglyceride values by 20, age was divided by ten, and we used the log (10) transformation of the weekly metabolic energy expenditure to approximate the normal distribution for this variable. We modeled systolic and diastolic blood pressure as continuous variables, with both centralized around their means.

**Figure 1:**
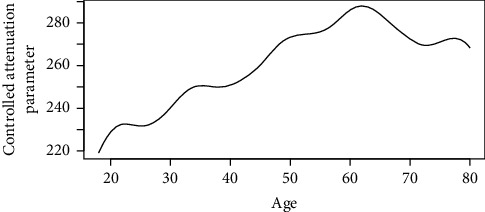
Loess smoother between age and controlled attenuation parameter.

**Figure 2:**
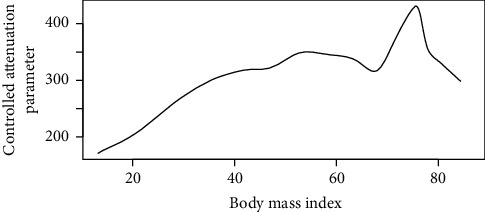
Loess smoother between the body mass index and the controlled attenuation parameter.

Though hemoglobin A1c is not part of the metabolic syndrome, we used it due to the lack of data on fasting blood sugar in the NHANES database. Also, our study focuses on identifying variables that are frequently used in clinical practice to identify patients at risk for liver steatosis or fibrosis. Therefore, we used body mass index instead of waist circumference. Also, these variables are highly correlated with each other and with liver steatosis, as shown in Figures 1S and 2S (supplementary material).

We used two other approaches to determine the predictors that substantially affect liver steatosis. In the first approach, we used the subset variable selection method using the RegSubsets package in R. In this method, the algorithm would select the model with the lowest Bayesian information criteria (BIC) and determine the effect of each predictor on reducing the BIC. In the second approach, we performed an adaptive Lasso regression. In contrast to the first approach, lasso regression penalizes the model while adding the covariates. In this approach, we ran an ordinary least squares model using all the predictors that were used in linear regression. Then, we used the inverse of these predictor coefficients as a penalty term in the 10-fold cross-validation to determine the optimal value of lambda. Then, we used the one standard error lambda value in lasso regression. This latter approach will shrink the coefficient parameter and drop the covariates contributing least to liver steatosis.

Also, we modeled the CAP and liver stiffness values (kPa) as binary outcomes using logistic regression models at the cutoff level of 294 dB/m for the former and 8.2 kPa for the latter, as recommended by Karlas et al. [6]. The same predictors used in the multivariable linear regression model were used in the multivariable logistic regression model with liver steatosis as an outcome. For the multivariable logistic regression model with liver fibrosis as an outcome, we only used the predictors that explains more than 5% of the model variance as determined by the gradient-boosted model (BMI, platelet count, hemoglobin A1c, and diastolic blood pressure), but we added the total weekly metabolic energy expenditure because it is a strong confounder and has a weak correlation with other predictors (no collinearity). Moreover, we calculated the predicted probabilities of developing liver steatosis among different values of predictors using the Naïve Bayes Classifier.

Finally, we used a decision tree algorithm to identify the appropriate cutoff of our continuous variables that best predict liver steatosis and fibrosis at 294 dB/m and 8.2 kPa, respectively. We used 10-fold cross-validation to identify the lowest value for the cross-validation error. Then we used the corresponding complex parameter (CP) in the decision tree model.

## 3. Results

Baseline characteristics for our population are shown in Table 1. The median age for our study was 38 (IQR 19–57). Males' and females' proportions were approximately equal. The median body mass index was 27, with a median metabolic energy expenditure of 1680 calories per week, and 32% of the participants had CAP values above 294 dB/m. It is worth mentioning that our data likely represent healthier people than those encountered in the hospital setting.

**Figure 3:**
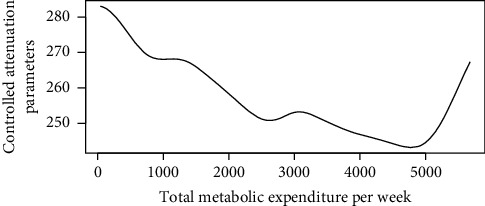
Loess smoother between total metabolic expenditure per week and the controlled attenuation parameter excluding the upper 0.25 quantile of the total metabolic energy expenditure per week.

**Figure 4:**
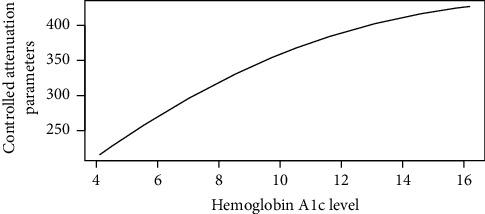
Loess smoother between hemoglobin A1c values and the controlled attenuation parameter.

**Figure 5:**
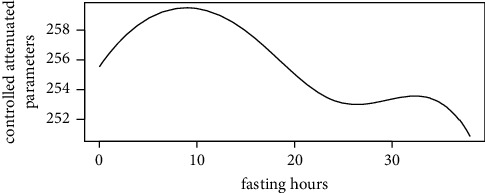
Loess smoother between fasting hours prior the controlled attenuation parameter testing.

**Figure 6:**
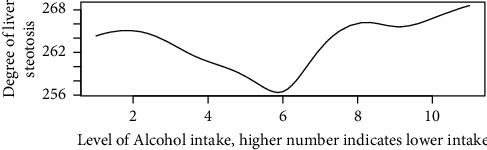
Loess smoother between the controlled attenuation parameter and the total monthly alcohol consumption; higher values on the *X* axis indicate lower total monthly alcohol consumption. (1) Drinks alcohol daily; (2) drinks alcohol almost daily; (3) drinks alcohol 3-4 times a week; (4) drinks alcohol 2 times a week. (5) Drinks alcohol once a week. (6) Drinks alcohol 2-3 times a month. (7) Drinks alcohol once a month. (8) Drinks alcohol 7–11 times a year. (9) Drinks alcohol 3–6 times a year. (10) Drinks alcohol 1-2 times a year. (11) Does not drink alcohol.

**Figure 7:**
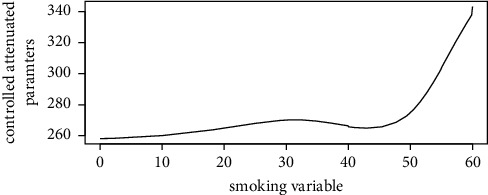
Loess smoother between the smoking intensity (average number of cigarettes smoked in the last month) and the controlled attenuation parameter.

**Figure 8:**
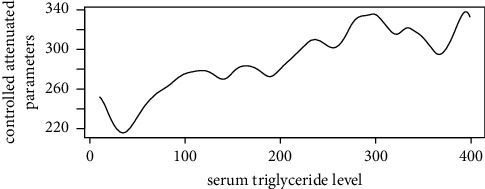
Loess smoother between the triglyceride values and the controlled attenuation parameter.

**Figure 9:**
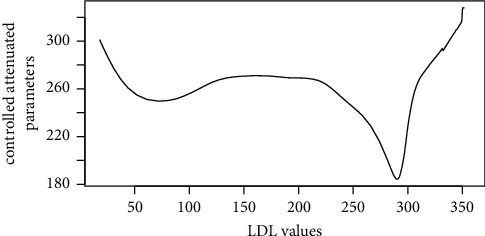
Loess smoother between low-density lipoprotein values and the controlled attenuation parameter.

**Figure 10:**
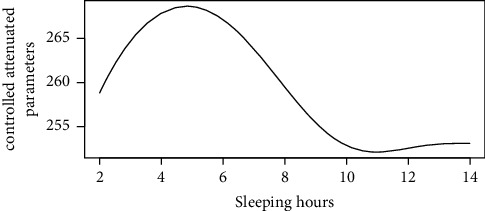
Loess smoother between sleeping hour values and the controlled attenuation parameter.

Figures 1–10 show our evaluation of the unadjusted linear association between each predictor and the controlled attenuation parameter. The linear assumption generally holds except for the sleeping, moderate level of alcohol consumption, and number of fasting hours before the procedure variables. For the sleeping variable, as shown in Figure 10, people who sleep more than 6 hours have lower values for the CAP compared to those who sleep less than 6 hours. The result of the univariate unadjusted model after creating a spline term after 6 hours is shown in Table 1S (supplementary material). People who sleep more than 6 hours have CAP values lower by 15 points compared to those who sleep less, with a statistically significant result. Similarly, we looked at the influence of hours of fasting before the procedure and the value of CAP. As shown in the Loess smoother and the result of univariable analysis for this model in Table 2S, only fasting more than 10 hours before the procedure is associated with a reduction in the CAP values with an average of 3.8 dB/m. These two variables were not included in the multivariable model because they are weak confounders and adding them would result in a wider confidence interval due to the decreasing number of participants with complete case analysis.

As shown in the Loess smoother for the other explanatory data analysis, the linear association between the predictor and the outcome did not hold when body mass index values above 45, triglyceride levels were above 1000 mg/dL, LDL values were above 300 mg/dL, and metabolic energy expenditure values were above 5730 METs/week. Also, these cutoffs were at or above the 0.95 quartiles for these covariates. Therefore, in our multivariable linear regression model, the analysis was limited at these cutoffs for these covariates, which will prevent overestimation or underestimation of the predictors' effect on liver steatosis or fibrosis. We decided not to include a smoother term or factor these predictors at these cutoffs due to the small number of subjects who have values above these cutoffs. Results for the multivariable linear regression model with liver steatosis as an outcome are shown in Table 2.

In this model, age, serum triglyceride level, and body mass index are strongly and significantly associated with the CAP. For each ten-year increase in age, there was a 4.46 increase in the CAP values. An increase in serum triglyceride level by 20 mg/dL was associated with a 2.5 increase in the CAP values, and an increase in the body mass index by one value was associated with 4.73 increase in the CAP values. On the other hand, females have 15.36 lower CAP values compared to males. For each one-point increase in the hemoglobin A1c value, there were 7.51 increments in the CAP values. For each 10-fold increase in the total metabolic energy expenditure per week, there was a 13.5 points reduction in the CAP values. Still, the results didn't reach the statistically significant cutoff of 0.05 for the latter two covariates. Also, we tested several other models with interaction terms. However, the variance analysis for these models was not significantly different from this model. The calculated *R*^2^ for this model was 51.3%.

To further test the robustness of our estimates, we evaluated the collinearity (correlation) among our predictors. Generally, there was a slight correlation among predictors except for a relatively strong correlation between hemoglobin A1c and serum triglyceride values, body mass index, and age, with a correlation of 0.29, 0.34, and 0.23, respectively, and between systolic and diastolic blood pressure with a correlation of 0.48. The correlation matrix was calculated after we replicated each subject in our dataset by its weight (Figure 1S). All results for the correlation structure were statistically significant at 0.05 except between metabolic energy expenditure and diastolic blood pressure and between platelet count and systolic blood pressure.  Figure 2S illustrates the correlation matrix among a subset of the predictors in addition to serum HDL and waist circumference. There was a strong negative correlation between HDL and triglyceride (−0.43), and the correlation between triglyceride and liver steatosis was slightly higher than HDL and liver steatosis (0.36 vs. −0.27). Therefore, we used serum triglyceride levels instead of serum HDL in the previously mentioned multivariable regression model. Waist circumference and BMI were strongly correlated with each other (0.92) and with liver steatosis (0.65 vs. 0.59, respectively), as shown in Figure 2S. Because BMI is a widely used and easily accessible clinical parameter, we used it in our models instead of waist circumference.

It's noteworthy to emphasise that regression analysis relies on complete case analysis. Data on liver steatosis and fibrosis were available for 5,948 participants, but only 1747 participants had complete data on all the variables that were used in the above linear regression model. However, if we exclude the metabolic energy expenditure (METs) variable (the predictor that has the largest number of missing values), 1894 participants will have complete data in all the variables. The results of the linear regression model excluding METs with liver steatosis as an outcome as shown in Table 6S. There was no significant difference in the coefficient's values between this model (Table 6S) and the one that included METs (Table 2). However, the *R*^2^ for the former analysis was 81%.

Our sensitivity analysis found that serum LDL, systolic and diastolic blood pressure, and smoking were not strongly associated with CAP values and were dropped out of the adaptive Lasso regression analysis (as shown in Table 4S). In the subset predictor selection algorithm, body mass index, serum triglyceride level, age, sex, hemoglobin A1c, and diastolic blood pressure achieved the model with the lowest BIC. In contrast, adding systolic blood pressure, moderate levels of alcohol consumption, smoking, metabolic energy expenditure, and serum LDL level results in increasing the value of the BIC (Figure 3S).

Table 3 illustrates the difference in baseline covariates between patients with CAP above and below 294 dB/m. The result reaches a statistically significant level for age, body mass index, metabolic energy expenditure, hemoglobin A1c, triglyceride level, and sex in univariate analysis.

The result of the multivariable logistic regression model using a CAP cutoff at 294 dB/m as an outcome and all other predictors as covariates is shown in Table 4. We excluded systolic blood pressure, diastolic blood pressure, serum LDL, and smoking from the multivariable model due to their poor correlation with the outcome of interest (they were dropped out of the adaptive lasso regression). We categorized our predictors into different strata for easy interpretability. Also, we reported adjusted relative risks (RR) among different predictors strata. Body mass index was the most clinically significant predictor for liver steatosis, with an adjusted RR of 3.91 for those with a BMI above 33 compared to those below 24. High serum triglyceride levels and diabetes are associated with an adjusted RR of 2. It is worth mentioning that the accuracy of the model using these predictors increases with a cutoff of 294 dB/m compared to 245 dB/m and an area under the receiver operator curve of 0.83 vs. 0.798, as shown in Figure 4S. Results were similar when we didn't exclude people in 0.95 quartiles or more on the predictor values, as shown in Table 3S.

We used the decision tree algorithm to identify the appropriate cutoff of our variables that best predict liver steatosis using 294 dB/m as a cutoff. The result of the decision tree is shown in Figure 5S. BMI and hemoglobin A1c are our decision tree's most important predictors for liver steatosis. Patients with a BMI above 30 and hemoglobin A1c above 5.7 have a 72% chance of liver steatosis compared to 14% among those with a BMI less than 30.

Finally, we looked at the probability of liver steatosis at 294 dB/m as a cutoff using a naïve Bayes classifier. 32% of our study population has liver steatosis (like decision tree analysis). Body mass index continues to be a strong predictor for liver steatosis in this method of classification as well. People with a body mass index of less than 24 have the lowest likelihood of liver steatosis at 0.4%. Patients with HBA1c above 5.8 have a high probability of liver steatosis, as shown in Figure 5SB. The accuracy of our Naïve Bayes' classifier was 78%. The results are shown in Table 5S.

Regarding liver fibrosis, in our decision tree analysis, when a liver stiffness value of 8.2 kPa was used as an outcome cutoff (advanced fibrosis), a body mass index at 41 was strongly predictable for liver fibrosis. Platelet counts strongly predict liver fibrosis among people with BMI less than 41, while age and serum LDL strongly predict liver fibrosis among those with BMI above 41, as shown in Figure 6S. Body mass index, platelet counts, and hemoglobin A1c and diastolic blood pressure are the most critical predictor in predicting liver fibrosis in the gradient-boosted model, as shown in Figure 7S, followed by AST/ALT ratio, age, and serum triglyceride level.

The baseline characteristics and the results of multivariable logistic regression models for liver fibrosis are shown in Tables 5 and 6, respectively. The multivariable logistic regression with liver stiffness at 8.2 kPa cutoff as an outcome, lack of sex, smoking, moderate level of alcohol consumption, serum LDL, systolic blood pressure, serum triglyceride, and age because these covariates are weak predictors, as shown in the gradient-boosted model (Figure 7S) (each account for less than 5% of the model variance).

In the multivariable linear regression model with liver fibrosis as an outcome and age, sex, hemoglobin A1c, serum HDL, body mass index, platelet counts and diastolic blood pressure as predictors (Table 7S). Sex, hemoglobin A1c, serum HDL at 50 mg/dL cutoff, body mass index, diastolic blood pressure, and AST/ALT ratio were statistically significant predictors for liver fibrosis. The *R*^2^ for this model was 20% (Loess smoother between HDL, body mass index, age, hemoglobin A1c and liver fibrosis are shown in Figures 8S–11S). In this model we centralized the continuous predictors around their means to make the interpretation for the intercept easier, but we dichotomized HDL at 50 mg/dL and platelets at 125 because participants with serum HDL below 50 had an increase in their liver stiffness values in our explanatory data analysis, as shown in Figure 8S and platelet value of 125 was identified as a significant cutoff in our regression tree.

Comparisons among different predictors between participants below and above the age of 50 are shown in Table 8S. We chose age 50 as a cutoff for these comparisons because participants above that age had a mean value of liver stiffness around 6 kPa.

## 4. Discussion

In the setting of a rising incidence of metabolic syndrome worldwide and in the United States, the incidence of NAFLD is expected to rise which could lead to liver fibrosis and subsequently cirrhosis in some patients (20–30% of those with NAFLD) [4]. Also, liver steatosis independently increases the risk for cardiovascular disease, type II diabetes, and chronic kidney disease and impairs the efficacy of medical therapy [4]. Therefore, it is important to correctly identify patients with NAFLD to provide an early intervention to prevent long-term cardiovascular and liver damage. However, there is debate on the best cutoff of the CAP and VCTE that has the highest accuracy in predicting liver steatosis and fibrosis, particularly among patients with NAFLD. Also, the interobserver variability between readings could reach upto 20 dB/ml for the CAP particularly among people with high a BMI in the absence of liver fibrosis, which could affect the steatosis classification. Moreover, liver steatosis could affect liver fibrosis measurement among patients with NAFLD [4, 8, 9]. Nonetheless, in the recent meta-analysis by Karlas et al., a cutoff of 294 dB/ml for the CAP had the highest accuracy in classifying NAFLD patients into those with and without liver steatosis, and an 8.2 kPa cutoff for liver stiffness had the highest accuracy in classifying these patients into those with <F2 vs. F3 and F4 fibrosis (advanced fibrosis) [6].

Using a large national database, we sought to determine the association between different clinical variables and the value of CAP and liver fibrosis. This would help general practitioners identify people at risk and whom to refer for liver steatosis and fibrosis screening. This is important, especially with the lack of serological tests that could early recognize NAFLD and the available serological tests for detecting liver fibrosis (AST to platelet ratio index (APRI), Fibrosis-4 score (FIB-4) that has aminotransferase levels, platelet counts and age as predictors, and nonaAlcoholic fatty liver disease fibrosis score (NAFLD fibrosis score) that has age, body mass index, blood glucose level, aminotransferase levels, platelet counts, and serum albumin as predictors) lack accuracy in predicting liver fibrosis compared to liver-related outcomes such as progression of the Model for End-Stage Liver Disease (MELD) or liver-related mortality [10].

Zenovia and colleagues evaluated the relationship between a variety of clinical parameters and liver steatosis and fibrosis. They concluded that body mass index, serum low-density lipoprotein, serum triglyceride level, fasting blood glucose, and serum uric acid correlate strongly with higher CAP. However, their study was limited by its small sample size [11]. In our multivariable regression model, we found that patients' age, body mass index, serum triglyceride level, and sex are significantly associated with CAP, while hemoglobin A1c and weekly metabolic energy expenditure are strongly associated with CAP with near statistically significant results at a *P* value cutoff of 0.05. In our analysis, the strength of association between hemoglobin A1c and liver steatosis increase slightly from 7.5 dB/m for one value increment in hemoglobin A1c to 9.4 dB/m after removing triglyceride level from the multivariable model due to the correlation among them (correlation of 0.29). On the other hand, serum LDL was not independently associated with liver steatosis. It's worth mentioning that serum HDL is highly and negatively correlated with serum triglyceride level and liver steatosis (−0.43, −0.27), as shown in Figure 2S but we used serum triglyceride level in our multivariable linear regression model with CAP as an outcome due to the higher correlation of triglyceride with CAP and to avoid collinearity.

In the unadjusted analysis, people who slept more than six hours had CAP fifteen points lower than their counterparts. The protective effect of sleep on liver steatosis is further supported by Mikolasevic et al. [12], who found a significant reduction in liver steatosis with more than 6 hours of sleep at night. Our univariate results further supported the Julio et al. study, which found that sleep of fewer than 6 hours is highly associated with cardiometabolic risk factors with an HR of 2.14 [8]. Adding hours of sleep to our multivariable regression analysis resulted in decreasing the total number of participants with complete case analysis and thus widening the confidence interval.

Body mass index was the strongest predictor for liver steatosis and fibrosis in our analysis (in regression tree analysis, the adaptive lasso regression for liver steatosis, and the gradient-boosted model for advanced liver fibrosis. Gupta et al. found that for each one-unit increase in body mass index above 23, there was a 19.6 times increase in the risk of hepatic steatosis in people above 50 years old [9]. Our data showed that patients with a body mass index of more than 34 and less than 45 had a relative risk of 4 for hepatic steatosis compared to those with BMI values less than 24 after adjusting for other confounders. Furthermore, our results were supported by Mjelle et al., who also found that CAP increases with increasing BMI values within the normal range with 4.4 dB/m for each 1 unit increase in BMI compared to 4.73 dB/m in our analysis [13]. In addition, our decision tree analysis showed that body mass index is the strongest parameter associated with liver steatosis and fibrosis. People with a BMI more than 30 and prediabetes have a 72% probability of liver steatosis, and those with a BMI above 30 and HBA1c less than 5.7 have a 43% probability of liver steatosis. On the other hand, our decision tree analysis using liver fibrosis as a binary outcome shows that people with a BMI more than 41 have a high probability of liver fibrosis (38%). Among those with a BMI below 41, platelet values could further determine the risk for liver fibrosis. The decision tree was able to detect interactions between BMI, platelet values, age, and serum LDL levels that were not detected in our regression model. Furthermore, this method is less affected by collinearity among predictors in contrast to regression analysis.

Hemoglobin A1c was strongly associated with liver steatosis and fibrosis in our multivariable regression analysis, Naïve Bayes analysis, decision trees, and gradient-boosted model. Patients with hemoglobin A1c of more than 5.8 but less than 6.5 had a 7% higher risk of developing liver steatosis after adjusting for other covariates in the multivariable analysis, as shown in Table 4. The increase in liver steatosis among patients with prediabetes was also reported by Naeem et al. study [14]. The result from the multivariable logistic regression model and the decision trees clearly shows that prediabetes is an independent risk factor for liver steatosis, especially in people with a body mass index above 30 (Figure 5S). On the other hand, hypertension particularly systolic blood pressure is poorly associated with liver steatosis, with results being removed from the adaptive lasso regression in addition to smoking and serum LDL values which suggest that these variables don't explain the variability in CAP values.

It is worth mentioning that 32% of our data has evidence of liver steatosis using 294 dB/m cutoff ultrasound criteria. Majelle et al. found similar findings, with 33% of the healthy cohort have evidence of liver steatosis using ultrasound criteria [13]. From the available literature, it appears that the accuracy of CAP in detecting hepatic steatosis is much lower in NAFLD compared to other etiology of hepatic steatosis such as hepatitis C. Furthermore, CAP appears to have higher accuracy in detecting higher stages of hepatic steatosis (>S1) with increasing the area under the ROC compared to the lower degree of hepatic steatosis (S0 vs. S1) as demonstrated in the individual-level meta-analysis by [6]. Nonetheless, the recent meta-analysis by Karlas et al. [6] concluded that 294 dB/m is the most accurate cutoff in identifying liver steatosis (S0 vs. S1–S3) among NAFLD patients to date.

In our logistic regression models using the linear form of our predictors (age, sex, serum triglyceride level, metabolic energy expenditure, moderate alcohol consumption, hemoglobin A1c, and body mass index) and CAP as an outcome, the AUC was slightly higher when we used a higher cutoff for the CAP (294 dB/m vs. 245 dB/m), as shown in Figure 4S. The increase in model accuracy at the 294 dB/m cutoff compared to 245 dB/m cutoff when including all the metabolic predictors suggests that the 295 dB/m cutoff is more accurate compared to lower cutoffs among patients with NAFLD which goes in hand with the recent Petroff et al. analysis.

Our results using multivariable linear, logistic regression, lasso regression, Naïve Bayes analysis, and regression trees show that body mass index and hemoglobin A1c, in addition to serum triglyceride level and age, are the main strong predictors in predicting liver steatosis, while smoking, systolic and, to a lesser extent, diastolic blood pressure and LDL values are weak predictors. On the other hand, BMI, hemoglobin A1c, platelet counts, diastolic blood pressure, and AST/ALT ratio, in addition to HDL at the 50 mg/dL cutoff, are the main predictors that predict liver fibrosis as demonstrated by gradient-boosted models, decision trees, and multivariable linear regression analyses (Figures 6S, 7S, and Table 7S, respectively).

In the linear regression model that has liver stiffness values as an outcome (Table 7S). Sex, hemoglobin A1c, serum HDL (at the 50 mg/dL cutoff), AST/ALT ratio, diastolic blood pressure, and platelet counts at the 125 cutoff are clinically and statistically significantly associated with liver fibrosis, with the strongest association for platelet counts at 125 cutoff, followed by AST/ALT ratio, sex, hemoglobin A1c, and HDL at 50 mg/dL cutoff and body mass index. On the other hand, and in contrast to liver steatosis, age was not significantly associated with liver fibrosis in the adjusted multivariable linear model, nor was it in the gradient-boosted model. It is notable that serum triglyceride level was strongly and statistically associated with liver steatosis and highly negatively correlated with serum HDL values. In contrast, serum HDL at 50 mg/dL cutoff appears to be strongly associated with liver fibrosis in the multivariable linear model, while serum triglyceride and LDL values were not and therefore were omitted from the multivariable linear model. Similarly, both variables were ranked low (7^th^ and 9^th^, respectively) in the gradient-boosted model. The lower number of participants with liver fibrosis (9% of our data population; 7% among those below the age of 50 and 12% among those more than 50 years old (Table 8S)) has limited the prediction of liver fibrosis in our models and could account for the mild discrepancy between the multivariable linear model, gradient-boosted model, and decision tree (age at 37 years old was identified as an important cutoff in determining the risk of liver fibrosis among people with BMI above 41, while age was not significant in the other two models).

Though our study used the same data used by Zhang et al. [7]. We used several machine learning algorithms to identify the predictors and their cutoffs that are strongly associated with liver steatosis and fibrosis. Therefore, our data provide valuable insight into stratifying the risk of liver steatosis and fibrosis among the general population. E.g., patients with a BMI less than 30 are less likely to have liver steatosis, while hemoglobin A1c helps further stratify the risk among those with a BMI over 30. Similarly, patients with a BMI less than 41 and a platelet count of more than 125 are less likely to have a significant degree of liver fibrosis. While those with a BMI of more than 41 have a 38% risk of liver fibrosis. Also, age and serum LDL and blood pressure further determine the risk of fibrosis among those with a BMI above 41.

Our results, particularly the decision trees, could help physicians identify people who should be referred for VCTE/CAP testing. Validating our finding of decision trees in prospective cohort studies with liver biopsy could help implement ultrasound-based screening for liver steatosis and fibrosis among NAFLD patients more cost-effectively.

The fibrosis-4 index for liver fibrosis (FIB-4 score) that depends on age, serum ALT, AST, and platelet count is widely used to identify people with liver fibrosis. However, the test has variable accuracy in predicting changes in liver fibrosis (0.65–0.81) and liver-related events (0.71–0.89). Also, if we used 3.25 as the cutoff, the test would have a high specificity of 0.92 but a low sensitivity of 0.51. The AST/platelet ratio index (APRI) seems to have poorer accuracy. The NAFLD-fibrosis score incorporates the patient's age, body mass index, and the presence or absence of impaired fasting glucose and albumin to the predictors of the FIB-4 score. Our results suggest that patients' body mass index, age, hemoglobin A1c, and platelet counts, LDL and HDL at the 50 mg/dL cutoff, and blood pressure are the most important predictors for predicting liver fibrosis in NAFLD patients. Therefore, our results suggest incorporating some adjustments to the parameter values for the NAFLD fibrosis score in addition to adding new parameters for the LDL, HDL, and blood pressure and modeling the hemoglobin A1c as a continuous variable instead of dichotomizing it. This could potentially increase the accuracy of the NAFLD score. This goes in hand with lee et al., who conclude that the NAFLD fibrosis score does not seem to provide more accuracy in predicting changes in liver fibrosis or liver-related events compared to the FIB-4 score alone [10].

Our study has some limitations: it mainly comprises relatively healthy volunteers, which is well demonstrated by the fact that the 95^th^ percentile of the BMI was 42.5, 7.2 for the hemoglobin A1c, and 171 for the LDL values. The absence of data on extreme values in our covariates has resulted in wider standard errors for our coefficient estimates in our models. Therefore, we highly recommend validating our decision tree results using data from patients who have metabolic syndrome, which will help in further accurately identifying people who would benefit most from ultrasound-based screening. Also, a significant number of patients had missing values on liver functions or platelet counts. Since machine learning algorithms rely heavily on complete case analysis and imputation methods could lead to unstable results, our results could be biased if patients with missing values had values different from their subgroups.

On the other hand, our study provides valuable insight into predictors that could be used to stratify the risk of liver steatosis and fibrosis and thus identify who could benefit most from screening. Moreover, some of our predictors, such as smoking, metabolic energy expenditure, hours of sleep, and fasting before the procedure, rely on people's reliability in providing accurate information. Informative bias could have resulted from differential recall errors between people with and without liver steatosis or fibrosis.

## Figures and Tables

**Table 1:** Baseline characteristics of our study population.

Patient characteristics	Median and interquartile range for continuous variables. Proportions and 95 confidence intervals for the categorical variables
Controlled attenuation parameter (mean, interquartile range^1^)	254 dB/m
Liver fibrosis (median, interquartile range^2^)	4.8 kPa
Age (median, interquartile range^3^)	38 years
Male (proportion, 95% CI^4^)	0.489
Body mass index (median, interquartile range^5^)	26.9
Metabolic energy expenditure per week (median, interquartile range^6^)	1680 kcal/week
Triglyceride levels (median, interquartile range^7^)	86 mg/dL
LDL levels (median, interquartile range^8^)	105 mg/dL
Hba1c (median, interquartile range^9^)	5.4%
Amount of alcohol drink in the last month^10^	Once a month
Systolic blood pressure (median, interquartile range^11^)	118 mmHg
Diastolic blood pressure (median, interquartile range^12^)	71 mmHg
Hours of fasting prior to the procedure (median, interquartile range^13^)	7 hours
Smoking^14^	Mean of 1.8 cigarettes in the last month (average cigarettes smoked for the last month)

^1^(212–302 dB/m), ^2^(4–5.9 kPa), ^3^(19–57 years), ^4^(0.95 CI = 47.2–51), ^5^(22.1–32.2), ^6^(240–5760 kcal), ^7^(57–132 mg/dL), ^8^(85–125 mg/dL), ^9^(5.2–5.8%), ^10^(two times a week-one to two times in the past year), ^11^(107–130 mmHg), ^12^(63–78 mmHg), ^13^(4–12 hours), ^14^(0.95 CI 1.48–2.16).

**Table 2:** Result of the multivariable linear regression model including the following covariates: LDL, triglyceride level, age, sex, hemoglobin A1c, total metabolic energy expenditure per week, body mass index, alcohol consumption, average number of cigarettes smoked per day for the last month, and systolic and diastolic blood pressure.

	Estimate	Std. error	*t* value	Pr (>|*t*|)
(Intercept)	125.65	29	4.36	0.012
LDL^*∗*^	−1.32	1.357	−0.972	0.39
Triglyceride^*∗*^	2.477	0.545	4.539	0.023
Age^*∗∗*^	4.455	1.463	3.046	0.034
Sex	−15.361	4.857	−3.162	0.0387
Hemoglobin A1c	7.514	2.733	2.75	0.0514
Metabolic energy expenditure^*∗∗∗*^	−13.459	5.36	−2.5	0.066
Body mass index	4.7306	0.3511	13.473	0.000176
Alcohol consumption^*∗∗∗∗*^	−0.923	0.798	−1.157	0.312
Smoking	0.4837	0.3478	1.391	0.24
Systolic blood pressure	2.824	1.852	1.52	0.20
Diastolic blood pressure	1.1	1.785	0.615	0.572

^*∗*^Values are divided by 20. Therefore, the estimated value reflects each 20-unit increase in the predictor value. ^*∗∗*^Values are divided by 10. Therefore, the estimate reflects each 10-unit increase in the predictor value. ^*∗∗∗*^value is the logarithm scale with base 10; therefore, the estimate reflects each 10-fold increase in the predictor value. ^*∗∗∗∗*^Alcohol consumption: 1, drinks alcohol daily; 2, drinks alcohol almost daily; 3, drinks alcohol 3-4 times a week; 4: drinks alcohol 2 times a week; 5, drinks alcohol once a week; 6, drinks alcohol 2-3 times a month; 7, drinks alcohol once a month; 8, drinks alcohol 7–11 times a year; 9, drinks alcohol 3–6 times a year; 10, drinks alcohol 1-2 times a year; and 11 does not drink alcohol.

**Table 3:** Baseline characteristics between people who had liver steatosis and those who did not using a cutoff value of 294 dB/m.

	≥294 dB/m	<294 dB/m	*P* value
Age (median, 95% CI)	52 years (49–54)	26 years (25-26)	<0.001
Body mass index (median, 95% CI)	34 (33-34)	24.5 (23.6–26.1)	<0.001
Metabolic energy expenditure (median, 95% CI)	1440 kcal/week (1184–1680)	1960 kcal/week (1820–2258)	<0.001
Hemoglobin A1c (median, 95% CI)	5.7 (5.7-5.8)	5.4 (5.3-5.4)	<0.001
Triglyceride (median, 95% CI)	117 mg/dL (109–128)	75 mg/dL (71–78)	<0.001
LDL (median, 95% CI)	113 mg/dL (104–119)	104 mg/dL (100–106)	0.026
Females (proportions, 95% CI)	41 (38–44)	54 (52–56)	
Alcohol intake	7 (7-8)	7 (6-7)	0.08
Hypertension (proportions, 95% CI)	0.25 (0.22–0.29)	0.13 (0.11–0.16)	<0.001

**Table 4:** The result of the multivariable logistic regression model with liver steatosis as a binary outcome using 294 dB/m as the cutoff.

Covariates	Coefficient	95% CI	*P* value	Adjusted relative risk	Standard error for the adjusted RR
Intercept	1.06	0.9034–1.243	0.52		
Age	1.0026	1–1.005	0.17		
Reference (10–57 mg/dL)					
Triglyceride (57–133 mg/dL)	1.172	1.06–1.29	0.033	1.85	0.375
Triglyceride (133–400 mg/dL)	1.247	1.1–1.40	0.021	2.18	0.467
Reference (40–720)					
Mets total B (720–2760)	0.901	0.846–0.960	0.032	0.722	0.0735
Mets total C (2760–5680)	0.927	0.810–1.06	0.33	0.797	0.177
Reference (male)					
Female	0.903	0.842–0.967	0.04	0.87	0.03
Hemoglobin A1c reference (4.1–5.2)					
Hemoglobin A1c B (5.2–5.8)	1.0187	0.911–1.139	0.76	1.066	0.21
(5.8–6.5)	1.069	0.945–1.211	0.35	1.24	0.26
(6.5–14.2)	1.320	1.183–1.472	0.008	1.99	0.344
Body mass index reference (15.7–24.1)					
Body mass index (24.1–33.6)	1.08	0.997–1.164	0.13	1.54	0.413
Body Mass index (33.6–45)	1.50	1.367–1.642	<0.01	3.91	1.06

**Table 5:** Baseline characteristics between people who had liver fibrosis and those who did not using a cutoff value of 8.2 kPa.

	≥8.2 kPa	<8.2 kPa	*P* value
Age (median, 95% CI)	55 years (44–57)	48 years (46–51)	<0.001
Body mass index (median, 95% CI)	36 (35–37)	27.5 (27-28)	<0.001
Metabolic energy expenditure (median, 95% CI)	1080 kcal/week (720–1440)	1920 kcal/week (1680–1987)	0.21
Hemoglobin A1c (median, 95% CI)	5.8 (5.7–5.9)	5.4 (5.4-5.4)	<0.001
Triglyceride (median, 95% CI)	105 mg/dL (99–117)	85 mg/dL (81–89)	0.011
LDL (median, 95% CI)	109 mg/dL (92–119)	105 mg/dL (101–109)	0.724
Females (proportions, 95% CI)	0.41 (0.36–0.47)	0.51 (0.49–0.54)	0.005
Alcohol intake	8 (7–9)	7 (6-7)	0.033
Hypertension (proportions, 95% CI)	0.27 (0.22–0.33)	0.16 (0.14–0.18)	<0.01

**Table 6:** Multivariable logistic regression model with liver fibrosis as an outcome using 8.2 kPa as cutoff.

Covariates	Coefficient	95% CI	*P* value
Intercept	1.12	1.07–1.17	0.007

Sex reference (male)	0.973	0.95–0.99	0.06

Hemoglobin A1c reference (4.1–5.2)			
5.2–5.8	1.02	1.004–1.033	0.06
5.8–6.5	1.08	1.04–1.13	0.021
6.5–14.2	1.2	1.15–1.28	0.002

Metabolic energy expenditure reference (40–720 kcal/week)			
720–2760 kcal/week	0.974	0.955–0.992	0.05
2760–5680 kcal/week	0.98	0.956–1003	0.16

Body mass index reference (15.7–24.1)			
24.1–33.6	0.996	0.97–1.02	0.73
33.6–45	1.15	1.1–1.2	0.004

Platelet count reference (8–209 cells/microliter)			
209–818	0.963	0.94–0.99	0.07

Diastolic blood pressure reference (54–63 mmHg)			
63–86 mmHg	0.98	0.943–1.01	0.3
86–133 mmHg	0.98	0.935–0.992	0.23

## Data Availability

The datasets generated for the current study are publicly available in the National Health and Nutritional Examination Survey database using the following web link: https://wwwn.cdc.gov/nchs/nhanes/search/datapage.aspx? Component=Examination&CycleBeginYear=2017.

## Supplementary Materials

Supplementary Materials A document entitled “Supplementary material” was added to our submission to avoid attaching more than 10 figures or tables in the main manuscript. The first figure (Figure S1) illustrates the correlation matrix between the predictors that were used in our analysis. The second figure (Figure S2) illustrates the correlation matrix among subset of predictors including serum HDL and waist circumference. The third figure (Figure S3) shows the reduction in the Bayesian information criteria while adding predictors to the model with Controlled Attenuation Parameter as an outcome. The fourth figure (Figure S4) illustrates the receiver operator curve for two logistic regression models at two different cutoffs of the Controlled Attenuation Parameter (294 vs. 245 dB/m). The fifth and sixth figures (Figures S5 and S6) illustrate the decision trees for liver steatosis at 294 dB/m and advanced liver fibrosis at 8.2 kPa as outcomes respectively. The seventh figure (Figure 7S) shows the relative importance of different predictors in predicting liver fibrosis using gradient boosted model (relative influence reflects the changes in model variance after imputing values of each predictor, a larger variance change indicates important predictor). Figures 8S–12S illustrates the loess smoother association between liver stiffness and serum HDL, hemoglobin A1c, body mass index and age respectively. Table 1S illustrates the linear association between liver steatosis and sleep using a spline term at 6 hours. Table 2S: illustrates the relationship between liver steatosis and hours of fasting prior to the procedure. Table 3S displays the result of logistic regression model using liver steatosis as an outcome and all predictors in our data without excluding people with extreme predictor values. Tables 4S displays the result of adaptive lasso regression for liver steatosis. Table 5S shows the result of naïve base classifier for the liver steatosis outcome at different predictor cutoff. Table 6S shows the result of multivariable linear regression model with liver steatosis as an outcome, and serum LDL, triglyceride, age, sex, hemoglobin A1c, body mass index, moderate alcohol consumption, smoking, systolic and diastolic blood pressure as predictors (excluding the metabolic energy expenditure to increase the number of participants with complete case analysis). Table 7S shows the result of linear regression model using liver stiffness as an outcome and age, sex, hemoglobin A1c, serum HDL at 50 mg/dL cutoff, body mass index, platelet counts at 125 cutoff, diastolic blood pressure, and AST/ALT ratio as predictors. Table 8S shows the adjusted comparison between participants above and below the age of 50 on different covariates.
