# Supplementary material for: Prediction of Liver Steatosis and Fibrosis Based on Clinical Variables Using a Large National Survey Database
Source: Can J Gastroenterol Hepatol. 2023 May 24;2023:1791500. doi: 10.1155/2023/1791500 (PMC10232144; doi:10.1155/2023/1791500)
Supplement: Supplementary Materials — A document entitled “Supplementary material” was added to our submission to avoid attaching more than 10 figures or tables in the main manuscript. The first figure (Figure S1) illustrates the correlation matrix between the predictors that were used in our analysis. The second figure (Figure S2) illustrates the correlation matrix among subset of predictors including serum HDL and waist circumference. The third figure (Figure S3) shows the reduction in the Bayesian information criteria while adding predictors to the model with Controlled Attenuation Parameter as an outcome. The fourth figure (Figure S4) illustrates the receiver operator curve for two logistic regression models at two different cutoffs of the Controlled Attenuation Parameter (294 vs. 245 dB/m). The fifth and sixth figures (Figures S5 and S6) illustrate the decision trees for liver steatosis at 294 dB/m and advanced liver fibrosis at 8.2 kPa as outcomes respectively. The seventh figure (Figure 7S) shows the relative importance of different predictors in predicting liver fibrosis using gradient boosted model (relative influence reflects the changes in model variance after imputing values of each predictor, a larger variance change indicates important predictor). Figures 8S–12S illustrates the loess smoother association between liver stiffness and serum HDL, hemoglobin A1c, body mass index and age respectively. Table 1S illustrates the linear association between liver steatosis and sleep using a spline term at 6 hours. Table 2S: illustrates the relationship between liver steatosis and hours of fasting prior to the procedure. Table 3S displays the result of logistic regression model using liver steatosis as an outcome and all predictors in our data without excluding people with extreme predictor values. Tables 4S displays the result of adaptive lasso regression for liver steatosis. Table 5S shows the result of naïve base classifier for the liver steatosis outcome at different predictor cutoff. Table 6S sho [file 1791500.f1.zip › Supplementary material(3Final_Yanal)_Yanal_submsission_Final (3).docx]

Supplementary material

Figure 1S: The correlation matrix between the continuous covariates that were included in the linear regression model. X indicates insignificant correlation P-value.

LUXCAPM=liver steatosis. LBXTR=Triglyceride level. LBDLDL=LDL level. RIAGEYR=age in year. BMXBMI=Body mass index. ALQ123=alcohol consumption (Higher numeric values indicate lower consumption). Metstotal=Total metabolic energy expenditure per week. LBXGH=hemoglobin A1c values. Smoke1=Average cigarettes smoked in the last month. Syst=Systolic blood pressure. Diast=Diastolic blood pressure

Figure 2S: The correlation matrix between the following covariates: LUXCAPM=liver steatosis. LBXTR=Triglyceride level. LBDLDL=LDL level. BMXBMI=Body mass index. LUXSMED=liver stiffness KPa. LBHD=serum HDL.BMXWAIST=waist circumference.


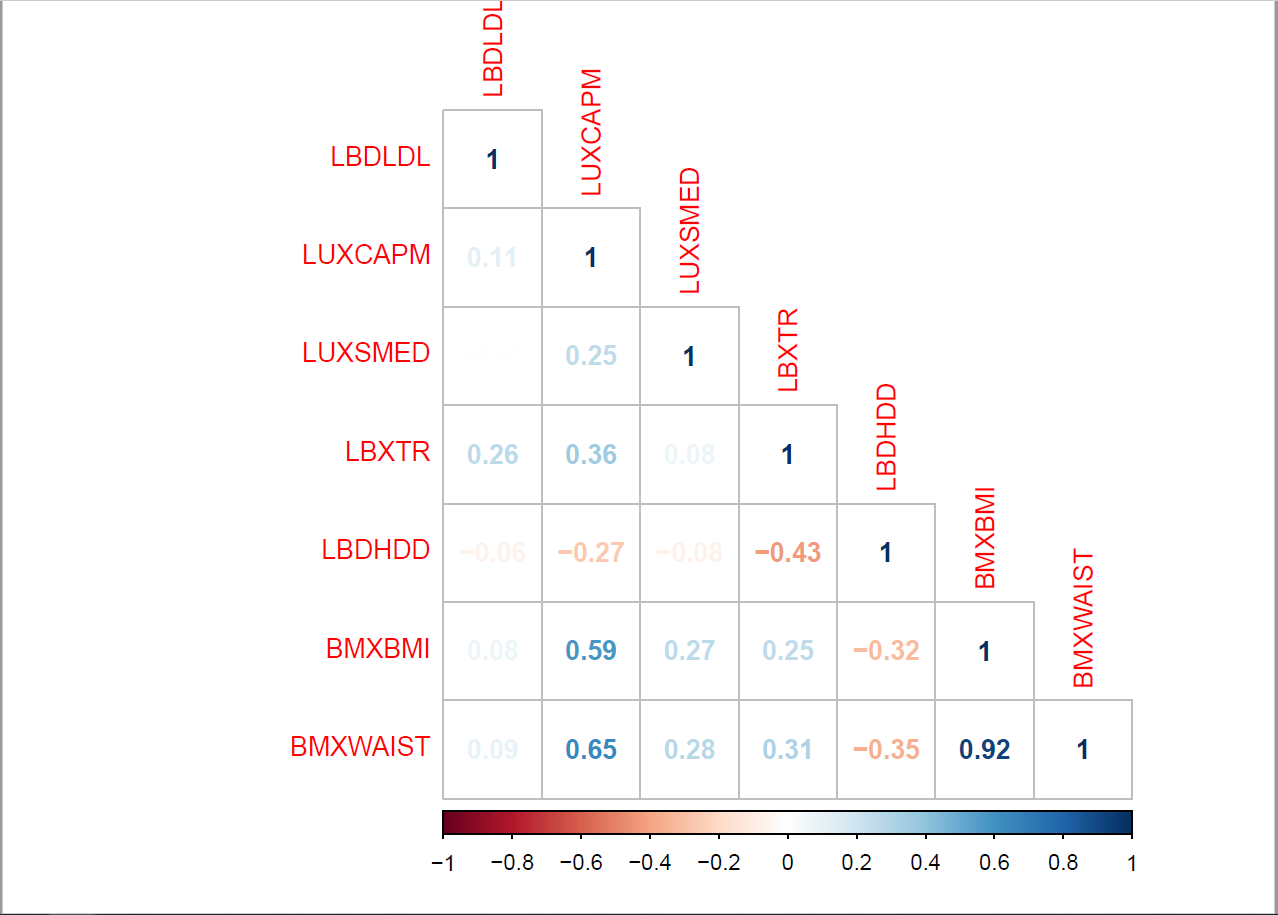


Figure 3S: Reduction in the Bayesian Information Criteria (BIC) using the subset variable selection method. Lowest value for the BIC was achieved by adding the following predictors: Body mass index, serum triglyceride level, age, sex, HbA1c and diastolic blood pressure. On the other hand, adding systolic blood pressure, alcohol consumption, smoking, metabolic energy expenditure and serum LDL level results in increasing the value for the BIC. Result was similar using forward selection method.

Figure 4S illustrates the Receiver Operator Curve (ROC) of two logistic regression models using two different cutoffs for liver steatosis on CAP (294 dB/m VS 245dB/m) using the same predictors on two models; age, gender, body mass index, serum triglyceride level, hemoglobin A1c, alcohol consumption and metabolic energy expenditure per week. The area under the curve was higher for the model with a cutoff value 294dB/m VS 245dB/m (0.8335 VS 0.798). ROC curves were created using Weighted ROC package to account for the weights in the survey design. TPR=True positive rate, FPR=False positive rate.

Figure 5S: Decision trees in predicting liver steatosis using 294 dB/m as cutoff. The following predictors were used in the regression trees: age, gender, body mass index, hemoglobin A1c, triglyceride level, serum LDL, weekly metabolic energy expenditure, history of smoking, systolic and diastolic blood pressure


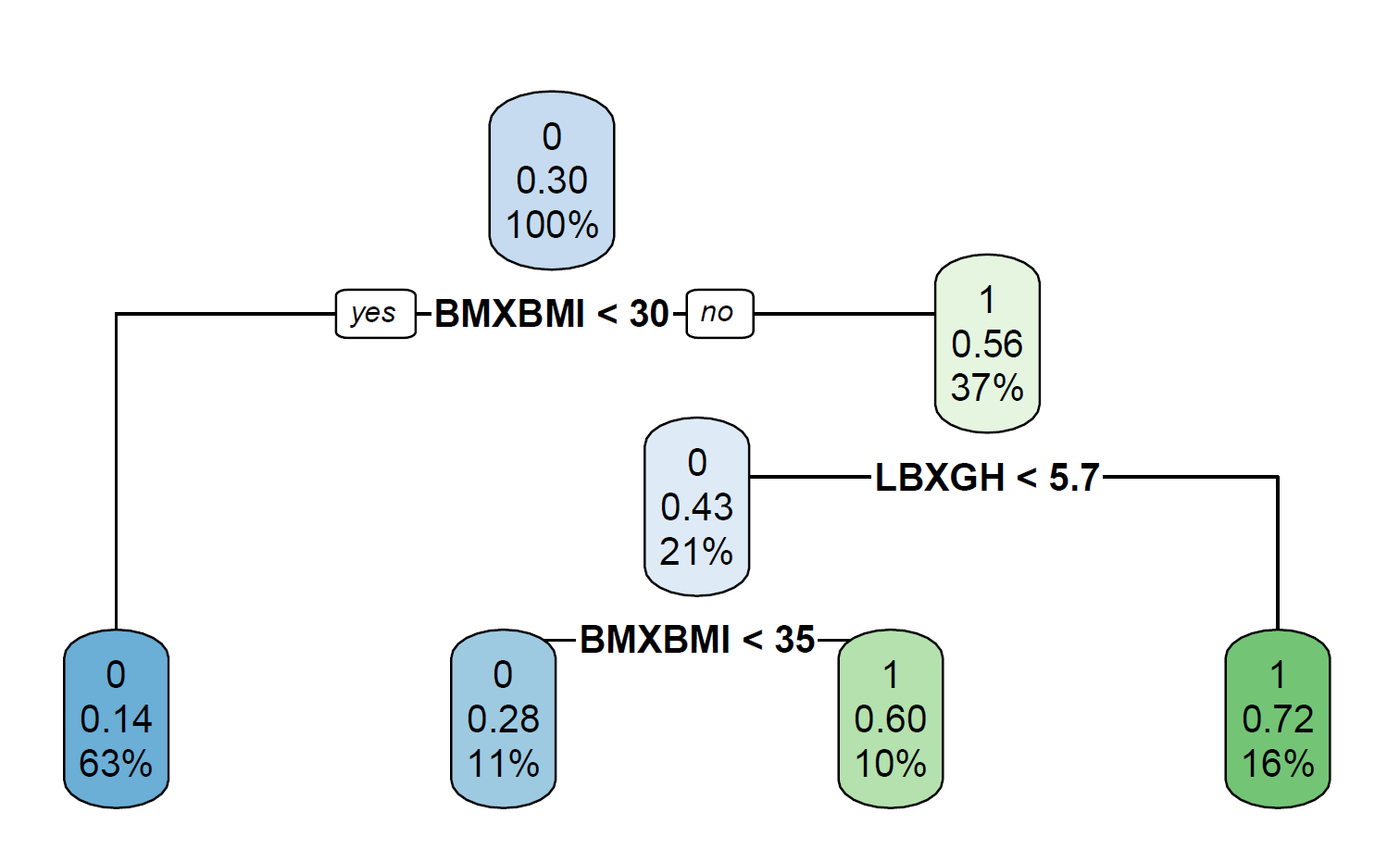


Figure 6S: Decision tree using liver fibrosis (stiffness) at 8.2 Kpa as an outcome, with body mass index, age, gender, hemoglobin A1c, platelet count, AST/ALT ratio, smoking, weekly metabolic energy expenditure, serum triglyceride and LDL level as predicators.

Figure7S: Illustrates the relative influence of the following variables (in descending order) on liver stiffness using the gradient boosted model (gbm). BMI, platelet counts, hemoglobin A1c, diastolic blood pressure, AST:ALT ratio, age, serum triglyceride level, systolic blood pressure, LDL, Alcohol consumption, metabolic energy expenditure, smoking and sex. The model was created using interaction. Depth of 6, shrinkage parameter of 0.001 and 3000 trees.

Figure 8S: Loess smoother between serum HDL and liver stiffness values.

Figure 9S: loess smoother between hemoglobin A1c and liver stiffness values

Figure 10S: loess smoother between Body mass index and liver stiffness values

Figure 11S: loess smoother between age and liver stiffness values

| Table 1S: Result of univariable linear regression analysis with CAP as an outcome and sleeping variable as a predictor using a spline term at 6 hours sleep | | | | |
| --- | --- | --- | --- | --- |
|  | Estimate | St. Error | t value | Pr(>\|t\|) |
| (Intercept) | 248.0928 | 12.337 | 20.111 | <0.01 |
| Sleep | 3.749 | 2.369 | 1.582 | 0.1396 |
| Sleep-spline1 | -15.253 | 5.901 | -2.59 | 0.024* |
| Sleep: sleep-spline1 | 0.7140 | 0.431 | 1.66 | 0.123 |

| Table 2S: Result of univariable linear regression analysis with CAP as an outcome and fasting hours as predictors using a spline term at 10 hours | | | | |
| --- | --- | --- | --- | --- |
|  | Estimate | Std. Error | t value | Pr(>\|t\|) |
| (Intercept) | 253.59 | 4.224 | 60.037 | <0.01 |
| Fasting | 1.033 | 0.5162 | 2.002 | 0.0684 |
| Fasting spline | -3.810 | 1.6797 | -2.27 | 0.043 * |
| Fasting: fasting spline | 0.0758 | 0.049 | 1.56 | 0.145 |
|  |  |  |  |  |

| Table 3S: The result of multivariable logistic regression model with liver steatosis as binary outcome using 294 dB/m as cutoff without excluding people with extreme values on predictors. | | | |
| --- | --- | --- | --- |
| Covariate | Coefficient | 95%CI | P-value |
| Intercept | 1.07 | 0.955- 1.2 | 0.36 |
| Age | 1.001 | 0.999-1.003 | 0.23 |
| Hemoglobin A1c Reference (3.8-5.2) |  |  |  |
| Hemoglobin A1c B (5.2-5.8) | 1.04 | 0.99 -1.11 | 0.27 |
| (5.8-6.3) | 1.24 | 1.15 -1.35 | 0.03 |
| (6.3-16.2) | 1.45 | 1.31 -1.60 | 0.03 |
| Male |  |  |  |
| Female | 0.92 | 0.89- 0.95 | 0.02 |
| Reference (10-57 mg/dL) |  |  |  |
| Triglyceride (57-132 mg/dL) | 1.11 | 1.05- 1.18 | 0.08 |
| Triglyceride (132-2684 mg/dL | 1.206 | 1.11- 1.32 | 0.05 |
| Body Mass Index  Reference (12.3-22.1) |  |  |  |
| Body Mass Index (22.1-32.5) | 1.07 | 1.03- 1.11 | 0.07 |
| Body Mass Index (32.5-86.2) | 1.518 | 1.44- 1.60 | <0.01 |
| Mets total A (0-1680) |  |  |  |
| Mets total B (1680-5760) | 0.951 | 0.90- 1.01 | 0.23 |
| Mets total C (5760-561144) | 0.957 | 0.90- 1.11 | 0.32 |
| Alcohol (1-7) |  |  |  |
| (7-10) | 0.995 | 0.93- 1.07 | 0.90 |
| (10-11) | 0.95 | 0.91- 0.99 | 0.13 |

| Table 4S: Result of adaptive Lasso regression analysis with liver steatosis as an outcome using 294 CAP as cutoff. Coefficients for serum LDL, systolic, diastolic blood pressure and smoking were shrunk to zero indicating their weak association with liver steatosis. | |
| --- | --- |
|  | s0 |
| (Intercept) | 41.1 |
| LDL | . |
| Serum Triglyceride level | 0.12 |
| Age | 0.49 |
| Gender | -15 |
| Metabolic energy consumption per week | -1.26 |
| Body mass index | 4.5 |
| Serum Alcohol level | -0.6 |
| Hemoglobin A1c | 4.5 |
| Systolic blood pressure | . |
| Diastolic blood pressure | . |
| Smoking | . |

Table 6S (A, B, C and D) illustrates the probabilities of having liver steatosis by the CAP using Naïve Bayes Classifier and 294 dB as cutoff. Table A shows the marginal probabilities, table B and C shows the conditional probabilities of having liver steatosis conditional on hemoglobin A1c, body mass index, metabolic energy expenditure per week and serum triglyceride respectively. The accuracy of this classification was 78%.

| Table 6SA: Marginal probabilities of liver steatosis | |
| --- | --- |
| Liver steatosis | Marginal probabilities |
| No | 0.69 |
| Yes | 0.32 |

| Table 5SB: Probabilities of liver steatosis conditional on different categories of hemoglobin A1c | | | | |
| --- | --- | --- | --- | --- |
| Liver steatosis | HbA1c 4.1-5.2 | 5.2-5.8 | 5.8-6.4 | 6.4-14.3 |
| No | 0.36 | 0.54 | 0.07 | 0.026 |
| Yes | 0.15 | 0.50 | 0.17 | 0.18 |

| Table 5SC: Probabilities of liver steatosis conditional on different categories of body mass index | | | |
| --- | --- | --- | --- |
| Liver steatosis | BMI (15.7-23.9) | 23.9-32.5 | 32.5-45 |
| No | 0.10 | 0.72 | 0.18 |
| Yes | 0.004 | 0.39 | 0.61 |

| Table 5SD: Probabilities of liver steatosis conditional on different categories of metabolic energy expenditure | | | |
| --- | --- | --- | --- |
| Liver steatosis | Metabolic expenditure (40-1680) | 1680-2880 | 2880-5680 |
| No | 0.40 | 0.32 | 0.31 |
| Yes | 0.50 | 0.24 | 0.26 |

| Table 5SE: Probabilities of liver steatosis conditional on different categories of serum triglyceride level. | | | |
| --- | --- | --- | --- |
| Liver steatosis | Triglyceride  (10-87) | Triglyceride  (87-132) | Triglyceride  (132-1000) |
| No | 0.32 | 0.48 | 0.20 |
| Yes | 0.08 | 0.50 | 0.43 |

| Table 6S: result of multivariable linear regression model using liver steatosis as an outcome and the following covariates: LDL, Triglyceride level, Age, Sex, Hemoglobin A1c, Body mass index, Alcohol consumption, average number of cigarettes smoked per day for the last month, Systolic and diastolic blood pressure, excluding the metabolic energy expenditure (MET). R2 for the model is 0.81 | | | | |
| --- | --- | --- | --- | --- |
|  | Estimate | Std. Error | t value | Pr(>\|t\|) |
| (Intercept) | 72 | 11.7 | 6.17 | 0.0016 |
| LDL* | -0.02 | 0.8 | -0.024 | 0.98 |
| Triglyceride* | 3 | 0.42 | 7.2 | 0.0008 |
| Age** | 5.6 | 0.94 | 6 | 0.00184 |
| Sex | -12.05 | 3.6 | -3.352 | 0.02 |
| Hemoglobin A1c | 8.1 | 2.1 | 3.93 | 0.011 |
| Body mass index | 4.34 | 0.25 | 17.66 | 0.0001 |
| Alcohol consumption**** | -0.863 | 0.527 | -1.51 | 0.40 |
| Smoking | 0.26 | 0.28 | 0.92 | 0.4 |
| Systolic blood pressure | 1.38 | 1.24 | 1.12 | 0.32 |
| Diastolic blood pressure | 2.3 | 1.15 | 2.1 | 0.093 |
| *Values are divided by 20. Therefore, the estimated value reflects each 20-unit increase in the predictor value. ** Values are divided by 10. Therefore, the estimate reflects each 10-unit increase in the predictor value. ^****^Alcohol consumption: 1: drinks alcohol daily, 2: drinks alcohol almost daily, 3: drinks alcohol 3-4 times a week, 4: drinks alcohol 2 times a week. 5: drinks alcohol once a week. 6: drinks alcohol 2-3 times a month. 7: drinks alcohol once a month. 8: drinks alcohol 7-11 times a year. 9: drinks alcohol 3-6 times a year. 10: drinks alcohol 1-2 times a year.11: doesn’t drink alcohol | | | | |

| Table 7S: Results of linear regression model with liver stiffness values as an outcome and the following covariates as predictors: Age, Sex, hemoglobin A1c, HDL, body mass index, platelet count, systolic blood pressure and AST/ALT ratio. * Predictors are centralized at their means. | | | |
| --- | --- | --- | --- |
|  | Estimate | St. Error | P-value |
| Intercept | 6.04 | 0.32 | <0.01 |
| Age* | 0.003 | 0.005 | 0.6 |
| Sex | -0.8 | 0.22 | 0.01 |
| Hemoglobin A1c* | 0.4 | 0.07 | 0.001 |
| HDL<50 mg/dL (Reference; HDL>=50 mg/dL) | 0.37 | 0.12 | 0.02 |
| Body mass index* | 0.24 | 0.02 | <0.001 |
| Platelet<125 (Reference; platelet counts >=125) | 11 | 4.5 | 0.05 |
| Diastolic blood pressure* | -0.02 | 0.007 | 0.05 |
| AST/ALT ratio * | 1.03 | 0.37 | 0.027 |

| Table 8S: Adjusted comparisons between people more than the age of 50 and those equal or less than 50 years-old in different variables (Liver stiffness, liver steatosis, serum triglyceride values, hemoglobin A1c, serum LDL, weekly metabolic energy expenditure (METs), platelet counts, systolic and diastolic blood pressure) | | | |
| --- | --- | --- | --- |
| Covariate | Age>50(mean, CI) | Age<=50 (mean, CI) | P-value |
| Liver stiffness values | 6Kpa (5.7-6.3) | 5.48Kpa (5.15-5.8) | 0.16 |
| Liver steatosis (mean)(SE) | 277dB/m (271-283) | 253dB/m (249-258) | 0.13 |
| Triglyceride | 113mg/dL (105-121) | 99mg/dL (94-105) | 0.73 |
| Hemoglobin A1c | 5.9% (5.8-6) | 5.38% (5.445-5.3) | <0.01 |
| LDL | 114 mg/dL (109-119) | 108mg/dL (105-111) | 0.14 |
| METs | 3456 Kcal/week (2927-3985) | 6339Kcal/week (5610-7068) | <0.01 |
| Platelet | 232 (223-242) | 245 (239-251) | 0.04 |
| Systolic blood pressure | 131 mmHg (129-133) | 116 mmHg (115-117) | <0.01 |
| Diastolic blood pressure | 73 mmHg (72-74) | 72 mmHg (71-73) | <0.01 |
