# Supplementary material for: Prediction of Liver Steatosis and Fibrosis Based on Clinical Variables Using a Large National Survey Database
Source: Can J Gastroenterol Hepatol. 2023 May 24;2023:1791500. doi: 10.1155/2023/1791500 (PMC10232144; doi:10.1155/2023/1791500)
Supplement: Supplementary Materials — A document entitled “Supplementary material” was added to our submission to avoid attaching more than 10 figures or tables in the main manuscript. The first figure (Figure S1) illustrates the correlation matrix between the predictors that were used in our analysis. The second figure (Figure S2) illustrates the correlation matrix among subset of predictors including serum HDL and waist circumference. The third figure (Figure S3) shows the reduction in the Bayesian information criteria while adding predictors to the model with Controlled Attenuation Parameter as an outcome. The fourth figure (Figure S4) illustrates the receiver operator curve for two logistic regression models at two different cutoffs of the Controlled Attenuation Parameter (294 vs. 245 dB/m). The fifth and sixth figures (Figures S5 and S6) illustrate the decision trees for liver steatosis at 294 dB/m and advanced liver fibrosis at 8.2 kPa as outcomes respectively. The seventh figure (Figure 7S) shows the relative importance of different predictors in predicting liver fibrosis using gradient boosted model (relative influence reflects the changes in model variance after imputing values of each predictor, a larger variance change indicates important predictor). Figures 8S–12S illustrates the loess smoother association between liver stiffness and serum HDL, hemoglobin A1c, body mass index and age respectively. Table 1S illustrates the linear association between liver steatosis and sleep using a spline term at 6 hours. Table 2S: illustrates the relationship between liver steatosis and hours of fasting prior to the procedure. Table 3S displays the result of logistic regression model using liver steatosis as an outcome and all predictors in our data without excluding people with extreme predictor values. Tables 4S displays the result of adaptive lasso regression for liver steatosis. Table 5S shows the result of naïve base classifier for the liver steatosis outcome at different predictor cutoff. Table 6S sho [file 1791500.f1.zip › Table 3S_Y.pdf]

Table 3S: The result of multivariable logistic regression model with liver steatosis as binary outcome using 294 dB/m as cutoff without excluding people with extreme values on predictors.			
Covariate	Coefficient	95%CI	P-value
Intercept	1.07	0.955- 1.2	0.36
Age	1.001	0.999-1.003	0.23
Hemoglobin A1c Reference (3.8-5.2)			
Hemoglobin A1c B (5.2-5.8)	1.04	0.99 -1.11	0.27
(5.8-6.3)	1.24	1.15 -1.35	0.03
(6.3-16.2)	1.45	1.31 -1.60	0.03
Male			
Female	0.92	0.89- 0.95	0.02
Reference (10-57 mg/dL)			
Triglyceride (57-132 mg/dL)	1.11	1.05- 1.18	0.08
Triglyceride (132-2684 mg/dL	1.206	1.11- 1.32	0.05
Body Mass Index Reference (12.3-22.1)			
Body Mass Index (22.1-32.5)	1.07	1.03- 1.11	0.07
Body Mass Index (32.5-86.2)	1.518	1.44- 1.60	<0.01
Mets total A (0-1680)			
Mets total B (1680-5760)	0.951	0.90- 1.01	0.23
Mets total C (5760-561144)	0.957	0.90- 1.11	0.32
Alcohol (1-7)			
(7-10)	0.995	0.93- 1.07	0.90
(10-11)	0.95	0.91- 0.99	0.13
